# Neurological Complications of Conventional and Novel Anticancer Treatments

**DOI:** 10.3390/cancers14246088

**Published:** 2022-12-10

**Authors:** Paola Alberti, Alessandro Salvalaggio, Andreas A. Argyriou, Jordi Bruna, Andrea Visentin, Guido Cavaletti, Chiara Briani

**Affiliations:** 1 School of Medicine and Surgery, University of Milano-Bicocca, 20900 Monza, Italy; 2 NeuroMI (Milan Center for Neuroscience), 20126 Milan, Italy; 3 Neurology Unit, Department of Neurosciences, University of Padova, 35131 Padova, Italy; 4 Neurology Department, Agios Andreas State General Hospital of Patras, 26335 Patras, Greece; 5 Neuro-Oncology Unit, Hospital Universitari de Bellvitge-ICO Hospitalet, Bellvitge Institute for Biomedical Research (IDIBELL), 08908 Barcelona, Spain; 6 Hematology and Clinical Immunology Unit, Department of Medicine, University of Padova, 35131 Padova, Italy

**Keywords:** chemotherapy-induced peripheral neurotoxicity, chemotherapy-induced peripheral neuropathy, chemotherapy-related cognitive impairment, chemofog, chemobrain, immune-checkpoint inhibitors neurotoxicity

## Abstract

**Simple Summary:**

Cancer survivors can experience neurological complications after exposure to anticancer therapy. Chemotherapy-induced peripheral neurotoxicity (CIPN), mainly consisting of sensory loss and neuropathic pain in hands and feet, is most commonly encountered. Cognitive impairment, although less frequent, is also a severe adverse event, significantly diminishing patients’ quality of life. The introduction of immunotherapy has resulted in durable remissions in several types of solid tumor malignancies, although severe neurological immune-related adverse events involving both the central and peripheral nervous system can occur in up to 10% of patients. We herein describe what it is currently known on the topic, and provide directions for future neuroprotection and symptomatic treatment studies.

**Abstract:**

Various neurological complications, affecting both the central and peripheral nervous system, can frequently be experienced by cancer survivors after exposure to conventional chemotherapy, but also to modern immunotherapy. In this review, we provide an overview of the most well-known adverse events related to chemotherapy, with a focus on chemotherapy induced peripheral neurotoxicity, but we also address some emerging novel clinical entities related to cancer treatment, including chemotherapy-related cognitive impairment and immune-mediated adverse events. Unfortunately, efficacious curative or preventive treatment for all these neurological complications is still lacking. We provide a description of the possible mechanisms involved to drive future drug discovery in this field, both for symptomatic treatment and neuroprotection.

## 1. Introduction

Awareness and knowledge of neurological side effects due to anticancer treatments has dramatically changed in the last decades. This is mostly due to the improvement in the prognosis of cancer patients, which has made long-lasting side effects, such as neurological ones, an increasingly less acceptable condition in cancer survivors, even in those who were treated in the adjuvant rather than in the metastatic setting. Thus, surveillance of patients’ quality of life (QoL) and prevention/mitigation of late/persistent toxicities has become part of the routine activities in daily oncological practice, taking into account also treatments other than conventional chemotherapies (e.g., hormonal treatments such as tamoxifen that can cause myalgia [1]) that last longer and have prolonged the period of observation of cancer survivors by the treating oncologist [2], thus increasing recognition of long lasting disturbances. Chemotherapy-induced peripheral neurotoxicity (CIPN) has attracted most of the clinical attention because it can be long-lasting, debilitating and poorly managed with available pharmacological approaches [3], thus exerting a significant social and economic burden [4].

In this review, we provide an overview of neurological complications after exposure to conventional chemotherapy and modern immunotherapies with immune checkpoint inhibitors (ICIs) and chimeric antigen receptor (CAR) T-cell treatments.

## 2. Chemotherapy-Induced Peripheral Neurotoxicity (CIPN)

### 2.1. Definition and Clinical Presentation

CIPN is a common adverse event of the most widely used anticancer drugs: taxanes, platinum drugs, epothilones, vinca alkaloids, proteasome inhibitors and thalidomide [5,6]. Scientific attention to CIPN has steadily increased over the last years. Doing a simple PubMed search for “[chemotherapy] and [neuropathy]” in the decade 1990–2000, only a quarter of the papers listed in the last 10 years can be found. Moreover, in both the American Society of Clinical Oncology (ASCO) and European Society of Clinical Oncology (ESMO) meetings, CIPN is currently a constant topic of discussion and dedicated educational sessions. At the institutional level, the FDA and National Cancer Institute (NCI) have organized two meetings focused on CIPN, namely, the NCI panel of the SxQoL Steering Committee Clinical Trials Planning Meeting in Chemotherapy Induced Peripheral Neuropathy: Developing Novel Trials Informed by Translational Science [7] and the Analgesic, Anesthetic, and Addiction Clinical Trial Translations, Innovations, Opportunities, and Networks (ACTTION) CIPN Trial Design [8].

CIPN, typically manifesting as a length-dependent sensory axonal polyneuropathy with evidence of numbness/paresthesia and/or neuropathic pain in a stocking and glove distribution, usually evolves during chemotherapy in a dose-dependent, cumulative manner. Severely affected patients are at increased risk of falls because of significant proprioception changes and sensory ataxia even in absence of major motor impairment. Actually, each drug class shows a separate clinical pattern, as summarized in Table 1. Impaired strength is not a prominent feature and it is generally rather mild and distal, as well as mostly associated with taxanes and vinca alkaloids. Neuropathic pain is instead far more frequent in patients treated with proteasome inhibitors, although pain has greatly decreased with subcutaneous administration [9]. Platinum compounds (and occasionally also thalidomide) are associated with the peculiar temporal pattern called coasting phenomenon: symptoms may worsen after chemotherapy completion/suspension [10]. Oxaliplatin is not only associated with a chronic, cumulative sensory axonal neuropathy but also with acute neurotoxicity. Even though this acute syndrome is transient and never dose-limiting, it represents a state of axonal hyperexcitability that is possibly linked to neuronal damage [10,11,12]. Acute manifestations are reported by nearly all treated patients since the first cycle and resemble the typical axonal hyperexcitability symptoms linked to channelopathies: transient cold induced paresthesia at limb extremities, cold-induced dysesthesia at oral cavity/pharynx, jaw spasm, cramps, lasting mainly 24–72 h after each oxaliplatin administration [13,14]. Autonomic dysfunction is most commonly seen with vinca alkaloids.

For hematological patients, in the last few years some adjustments of treatment schedules have contributed to reduce CIPN associated with thalidomide and bortezomib. Novel drugs, including lenalidomide [15] and pomalidomide [16], have been introduced as an alternative to thalidomide, as they are associated with less common and less severe neurotoxicity. Likewise, the subcutaneous administration of bortezomib reduced the rate of CIPN development [9].

**Table 1:** CIPN clinical presentation for the different drug classes.

Drug Class	Clinical Presentation
**Platinum drugs—cisplatin and carboplatin**	Distal, symmetric, upper- and lower limb impairment/loss of all sensory modalities. Sensory ataxia and gait imbalance are frequent. Early reduction/loss of DTR. Coasting phenomenon. Carboplatin-related CIPN is usually milder.
**Platinum drugs—oxaliplatin (acute)**	Lasting 24–72 h after each administration. Cold-induced transient paresthesia at limb extremities, head and neck region (e.g., mouth, pharynx). Cramps/muscle spasm in throat muscle, jaw spasm, fasciculations
**Platinum drugs—oxaliplatin (chronic)**	Similar to cisplatin.
**Taxanes**	Distal, symmetric, upper and lower limb impairment/loss of all sensory modalities. Gait unsteadiness due to sensory ataxia. Distal, symmetric weakness in lower limbs is generally mild. Myalgia syndrome is frequent (as an atypical neuropathic pain?). Reduction/loss of DTR.
**Epothilones**	Similar to taxanes, but neuropathic pain is less frequent.
**Vinca Alkaloids**	Distal, symmetric, upper and lower limb impairment/loss of all sensory modalities. Neuropathic pain/paresthesia at limb extremities is relatively frequent. Distal, symmetric weakness in lower limbs progressing to foot drop. Autonomic symptoms (e.g., orthostatic hypotension, constipation) are more frequent than with other drug classes. Reduction/loss of DTR.
**Bortezomib**	Mild to moderate, distal, symmetric loss of all sensory modalities occurs. Neuropathic pain is frequent and often even severe. Mild distal weakness in lower limbs is possible. Autonomic symptoms (e.g., orthostatic hypotension, constipation). Reduction/loss of DTR.
**Thalidomide and analogues**	Relatively frequent neuropathic pain at limb extremities. Mild to moderate, distal, symmetric loss of all sensory modalities. Weakness is rare. Reduction/loss of DTR. Lenalidomide and pomalidomide are associated with a less severe neurotoxicity profile. Coasting phenomenon **

DTR: deep tendon reflexes. ** Described mainly in patients treated for systemic lupus erythematosus [17,18].

Another crucial aspect to consider is that different chemotherapies may induce diverse phenotypes of neurotoxicity in terms of acute or chronic constellation of symptoms: they can be with or without pain, and with various activity limitations as well as need for chemotherapy modification [19]. The impact of CIPN on patients’ QoL is now well recognized, although there is still some difficulty in understanding the full spectrum of clinical signs and symptoms. For instance, QoL impairment reported by individual subjects is frequently attributed to pain, but in most cases this is due to a sum of symptoms and signs without any real pain, but still severely invalidating (e.g., impaired equilibrium, altered sensory perception, difficulty in manipulating small objects). The erroneous interpretation of CIPN features might have contributed to the failure of trials on CIPN, designed mainly as pain trials, such as in the case of antidepressant and antiepileptic drugs used as possible neuroprotectants and not as symptomatic agents [20,21].

Moreover, it should always be considered that patients and health-care professionals may have very different perception of CIPN and it is likely that subjects undergoing chemotherapy tend to under-report CIPN symptoms, mostly because they fear receiving less-effective or reduced-dose chemotherapy regimens [22].

### 2.2. Mechanisms

The full spectrum of CIPN mechanisms is not yet totally known, but bench-side studies have shed light on potential neurotoxicity and axonal damage pathways. Since anticancer drugs have a different cytotoxic action against cancer cells, it should be expected that different neurotoxicity mechanisms are involved in CIPN, according to different drugs. Platinum drugs form interstrand DNA adduct [23] leading to cell cycle arrest in cancer cells [24], and acquainted/activated platinum complexes are able to bound crucial cytoplasmic nucleophiles (glutathione, methionine, metallothioneins and cysteine enriched-proteins) depleting cancer cells of antioxidants and thus favoring lipids and proteins peroxidation [25]. Several other classes, instead, such as taxanes, epothilones and vinca alkaloids, target microtubules blocking cancer cells in metaphase. Taxanes and epothilones alter microtubules depolymerization hyperstabilizing microtubules and act on tubulin dimers to allow polymerization in absence of guanosine triphosphate (GTP) and other microtubules-associated proteins [19]. On the contrary, vinca alkaloids promote microtubule depolymerization, forming a stable complex in the GTPase tubulin domain preventing GTP hydrolysis [6]. Proteasome inhibitors (e.g., bortezomib) are able to block the degrading system, causing protein accumulation, which then leads to apoptosis [26].

In neurons, drugs targeting microtubules—taxanes, epothilones, vinca alkaloids—can alter axonal transport, as demonstrated by several in vitro experiments [27]. Axonal transport was shown to be disrupted also by bortezomib [28,29], and a recent detailed in vitro/in vivo study elucidated the possible connection between proteasome inhibition and altered axonal transport by observing that proteasome inhibition caused delta 2 tubulin accumulation, thus undermining microtubule stability and dynamics with consequent axonopathy and altered mitochondria motility [30]. Altered axonal transport might also play a role in platinum drugs neurotoxicity, as suggested by neurographic in vivo molecular imaging [31], even though it is still to be verified if disrupted axonal transport is an early or late event in the mechanisms of damage.

Mitochondrial dysfunction might also play a role as suggested by morphological alterations after exposure to anticancer drugs, such as swelling, vacuolization, enlargement and loss of cristae structure [27]. Platinum drugs alter mitochondrial DNA since it does not benefit from surveillance of DNA repair systems as it is for nuclear DNA [32]. Vinca alkaloids and taxanes are associated with alterations in mitochondrial fission/fusion processes [27], and bortezomib with impaired mitochondrial calcium (Ca^2+^) homeostasis and respiratory chain failure [33]. Ca^2+^ overload is toxic to neurons since it activates Ca^2+^-sensitive calpain, phospholipases, and nitric oxide [34].

Oxaliplatin-related acute neurotoxicity syndrome is a specific and peculiar one that resembles genetic diseases affecting voltage-operated ion channels (VOC) [10]. Adelsberger et al. [35] individuated, via path-clamp studies, sodium-VOC (Na-VOC) as the main target of oxaliplatin acute neurotoxicity; Groulleau et al. [11], as well as Webster et al. [36] provided similar observations. In line with this, a study comparing wild-type and Scn8a^med^ mice (i.e., animals lacking NaVOC 1.6) showed that Scn8a^med^ mice did not develop alterations in neuronal excitability [37], and this was later confirmed by another study in which Nav1.6 blockers were able to decrease oxaliplatin-related hyperexcitability in animals [38]. Other authors also suggested that potassium VOC might also play a role [39,40,41,42]. Intriguingly, this transient unbalance in axonal excitability was recently linked to axonal damage development, suggesting that acute and chronic oxaliplatin-related neurotoxicity can be related (i.e., the former predisposing to the latter), as previously suggested by clinical data [43]. An unbalance in NaVOC can lead to a sustained depolarization, which can activate the reverse mode of the sodium/calcium exchanger 2 (NCX2), thus resulting in toxic Ca^2+^ accumulation and axonal damage [44]. In line with this, it was demonstrated that preventing acute oxaliplatin-related neurotoxicity with a NaVOC modulator completely prevented CIPN development in a rodent model [45]. Therefore, targeting acute oxaliplatin neurotoxicity might be a feasible option to prevent the full expression of the chronic manifestations too.

In the last few years, a more common general pathway was also considered as potentially pivotal in CIPN pathogenesis: the sterile-α and Toll/interleukin 1 receptor (TIR) motif containing protein 1 (SARM1); this NAD-degrading protein participate in events leading to Wallerian degeneration [46], as confirmed by the fact that SARM1-deficient mice are protected against axonal damage [47]. In CIPN setting, SARM1 knockout mice showed promising neuroprotection evidence against paclitaxel [48], vincristine [49], and oxaliplatin neurotoxicity [50].

Another general mechanism to be considered, as extensively addressed by Fumagalli et al. [51], is the potential involvement of neuroinflammation in axonal damage, as suggested by preclinical studies (rodent models): CIPN development can be mitigated modulating neuroinflammation [52,53,54].

For thalidomide, the exact mechanisms of neurotoxicity are yet to be fully elucidated, but the antiangiogenic effect and the decreased VEGF were suggested to be pivotal [6].

### 2.3. Assessment and Therapeutic Approach/Strategy

So far, there is no consensus on the gold standard to detect, monitor and grade CIPN. However, over the last two decades, many efforts have been made by large international study groups to solve this unmet clinical and scientific need. Physicians’ and patients’ CIPN perceptions and its impact on QoL are different, yet complementary [55]. Therefore, the best way to evaluate CIPN is a combination of clinician-reported outcomes (CROs) and patient-reported outcomes (PROs). This is in line with the indications given by the already mentioned ACTTION working group and the Consortium on Clinical Endpoints and Procedures for Peripheral Neuropathy Trials (CONCEPPT) working group, both composed by neurologists, oncologists, pharmacists, clinical trialists, statisticians, and regulatory experts [8]. Among PROs, the FACT/GOG-NTX and the European Organization for Research and Treatment of Cancer (EORTC) CIPN20 [7] have gained the most widespread use, while among CROs, the Total Neuropathy Score (© Johns Hopkins University; TNS) or one of its versions such as the TNSc (TNS clinical) showed valid clinimetric properties [56]. Notably, the widely used scale in oncology clinical trials, the NCI-CTCAE, raised concerns for appropriateness for CIPN assessment [57]. Moreover, quite recently even stronger indication on the use of PROs and CROs to detect CIPN has emerged: for the first time in CIPN setting, responsiveness and minimally clinical important difference was demonstrated for FACT/GOG-NTX and TNSc, as well as for a shorter version of both of them; in particular, the reduced TNS-nurse version (TNSn) was found as a reliable alternative if a formal neurological examination is difficult to be performed [58].

Apart from grading scales, there are other potentially valuable tools that have been explored to detect CIPN. Nerve conduction studies (NCS) can be an ideal objective measure to detect the length-dependent CIPN [59], in particular the testing of the most distal branches of the peripheral nervous system, such as the dorsal sural nerve [60]. However, it should be noted that NCS are able to detect only damage of large myelinated fibers, lacking to demonstrate small fiber involvement [61]. Neuroimaging techniques, including ultrasound and magnetic resonance neurography, hold promise for monitoring CIPN, although the evidence is still rather limited [62,63].

Serum biomarkers were also investigated to detect and monitor CIPN, despite none yet achieving entry into routine clinical practice. So far, the most promising blood biomarker assay is neurofilament testing. In particular, neurofilament light chain (NfL) might be of interest since they are released in interstitial fluids when axonal damage occurs [64], and were tested in various neurological disturbances affecting both central and peripheral nervous systems [64,65,66,67,68,69,70,71,72,73]. In CIPN, NfL levels were initially tested in animal models with promising results [10,19,68] and subsequently explored in small clinical studies. Karteri et al. [74] prospectively followed paclitaxel-treated patients demonstrating a significant longitudinal increase in NfL levels that were also significantly more increased in patients most severely affected. Huehnchen et al. [75] showed similar data in paclitaxel-treated patients. Based on these preliminary results, upon validation in a large clinical validation study, NfL might be qualified to enter clinical practice in the coming years.

With regard to CIPN treatment, unfortunately, no evidence of a preventive and/or curative treatment is available. As recently addressed by a meta-analysis performed by the ASCO [76], there was only a modest recommendation for the use of duloxetine in the symptomatic treatment of CIPN, while none of the several neuroprotective agents tested thus far have achieved significance to be recommended in CIPN prophylaxis. To overcome this, it is advised to design future neuroprotection clinical trials on a strong biological rationale using the most solid and robust outcome measures, as described above, to detect and grade CIPN.

## 3. Novel Anticancer Drugs and Peripheral Neurotoxicity

During the last decades several efforts have been made to decrease the use of chemotherapies and to develop more selective agents targeting key molecules involved in the pathogenesis of solid cancers or hematological malignancies. These novel classes of drug include monoclonal antibodies (mAb), usually human or chimeric IgG antibody targeting a cancer-antigen; drug conjugated antibodies, that is a mAb conjugated with a chemotherapy agent; small molecule inhibitor or chimeric antigen receptor T lymphocytes. Different from standard anticancer drugs, their peripheral neurotoxicity has not been diffusely investigated in clinical studies, and peripheral neurotoxicity data are mainly based on information gained in oncological clinical trials whose primary aim was to demonstrate the efficacy of the specific novel drug, with little attention to peripheral neurotoxicity.

### 3.1. Conjugated Monoclonal Antibodies

In this class of drugs, the mAb is usually combined with a microtubule inhibitor, such as brentuximab vedotin and polatuzumab vedotin.

The most studied is brentuximab vedotin which is approved for the treatment of advanced stage and relapsed/refractory cases of Hodgkin lymphoma, anaplastic large-cell non-Hodgkin lymphoma and Sezary syndrome. Brentuximab is an anti-CD30 monoclonal antibody conjugated with monomethyl auristatine E, which is a microtubule inhibitor. Sensory symptoms are more common than motor, with numbness, paresthesia and tingling being the most frequently experienced symptoms. Neuropathic pain develops in almost half of patients [77]. NCS confirm sensory axonal neuropathy in most of the cases. Electron microscopy examination revealed alterations in axonal cytoskeleton and altered orientation of microtubules [78,79]. In a pivotal phase 2 trial brentuximab at 1.8 mg/kg every 3 weeks up to 16 doses, was given as single agent to relapsed patients (*n* = 102) [80]; after a median follow-up of more the 5 years, 55% of enrolled patients experienced neuropathy symptoms, most (88%) experienced resolution or improvement. Of the 15/102 patients with ongoing neuropathy at last follow-up, 11 patients had grade 1 severity and 4 patients had grade 2. In the phase 3 Echelon-1 trial, brentuximab vedotin was given at 1.2 mg/kg every 2 weeks for 12 doses in the frontline setting in combination with AVD (doxorubicine, vinblastine and dacarbacinze) [81]. Any-grade peripheral neuropathy occurred in 67% patients in the A+AVD group and in 43% of patients in the ABVD group. At 5 years, 19% of patients in the A+AVD group and 9% in the ABVD group had ongoing peripheral neuropathy. Most of these cases were mild with only 3% of grade 3 or 4 neuropathy in both arms. In the majority of patients, the neuropathy completely resolved (85% A-AVD) or improved (13% A-AVD) after the end of therapy with an estimated time to resolution of 34 weeks for A-AVD compared to 16 weeks after ABVD treatment.

Polatuzumab vedotin is an anti-CD79b mAb conjugated through a protease-cleavable linker to monomethyl auristatin E which has recently been approved in the treatment of relapsed diffuse large B cell non-Hodgkin lymphoma. Polatuzumab is given at 1.8 mg/kg every 3 weeks in combination with bendamustine and rituximab. In the phase 2 trial, 31% of patients developed peripheral neuropathy with only 2% of grade 3 or 4. Most common symptoms were sensory neuropathy, muscular weakness, paresthesia, muscle atrophy, hypoesthesia, gait disturbance, decreased vibratory sense, hypotonia and neuralgia. (bloodadvances.2021005794). Polatuzumab vedotin has also been investigated in the frontline setting in combination with CHP (cyclophosphamide, doxorubicin and prednisone) compared with the standard of care, which is R-CHOP [82]. Peripheral neuropathy of any grade was reported in 53% of patients who received Pola-R-CHP and in 54% of those who received R-CHOP, (grade 2 or higher was reported in 13.8% and 16.7% of the patients, respectively). The median time to the onset of any neuropathy was 2.3 months in the pola-R-CHP group and 1.9 months in the R-CHOP group. The median time to resolution of the neuropathy was 4.0 months and 4.6 months, respectively. Very few patients discontinued any treatment because of peripheral neuropathy. The percentage of patients who had peripheral neuropathy that led to dose reduction was lower among those who received polatuzumab vedotin than among those who received vincristine (4.4% vs. 8.0%) [82].

### 3.2. Small Molecule Inhibitors

This class of drug targets kinases or proteins that play a pivotal role in the pathogenesis of cancer.

Bruton’s tyrosine kinase (BTK) is a downstream kinase of the B-cell receptor signaling. The first in class BTK inhibitor, ibrutinib, is widely used in the treatment of chronic lymphocytic leukemia, mantle cell lymphoma, marginal zone lymphoma and Waldenström’s macroglobulinemia. Despite patients with chronic lymphocytic leukemia and Waldenström’s macroglobulinemia might experience disease-related neuropathy [83,84,85], ibrutinib-induced neuropathy has also been reported. In a phase 2 study in relapsed-refractory patients, 13% developed any grade neuropathy but 0.5% was of grade 3 or 4. However, ibrutinib showed also to improve neurological symptoms in patients with Waldenström’s macroglobulinemia. Nine patients treated for IgM-related demyelinating neuropathy (3 with anti-MAG antibody neuropathy) who experienced disease progression or were refractory to rituximab showed stabilization or improvement of their sensory neuropathy with ibrutinib [86]. Ibrutinib was also investigated in 3 patients with Waldenström’s macroglobulinemia and anti-MAG antibody neuropathy. All the 3 patients reported an early and subjective benefit, consistent with the objective improvement, especially of the sensory symptoms [87]. Second-generation BTK inhibitors, acalabrutinib and zanubrutinib, have become available but no drug-induced peripheral neuropathy was reported [88,89].

Other two well explored classes of drug are PI3K inhibitors [90] and BH3-mimetic [91]. However, drug-induced peripheral neuropathy has not been reported. Noteworthy, it has been recently published a case of relapsed anti-MAG neuropathy successfully treated with venetoclax, a BCL-2 inhibitor [92].

### 3.3. Immune Checkpoint Inhibitors (ICI)

Tumor cells are known to be surrounded by reactive non-neoplastic cells that are unable to recognize and kill cancer cells due to the deregulation and overexpression of immune checkpoint proteins such as PD-L1, CTLA4, TIM-3, LAG3. Binding of antigen expressed by neoplastic cells with a receptor expressed on CD4 and/or CD8 T lymphocytes inhibits most immune responses. mAbs binding antigen or receptor are able to drive healthy T lymphocytes to recognize and kill tumor cells. In the last years ICI have become the standard of treatment in several solid cancers and Hodgkin lymphoma. These mAbs have been associated with a high incidence of immune-mediated adverse events, as it is discussed in the next section.

Chimeric antigen receptor T lymphocytes (CAR-T) are a novel and fascinating tool that allow in vitro engineered patient-derived T lymphocytes to express new receptors able to bind cancer cells. CAR-T therapies (tisagenlecleucel, axicabtagene ciloleucel, brexucabtagene autoleucel, lisocabtagene maraleucel, idecabtagene vicleucel) are currently approved for diffuse large B cell lymphoma, mantle cell lymphoma and acute lymphoblastic leukemia, but are currently under investigation also in solid cancers. However, major limitations to CAR-T cell therapy remain, including severe, life-threatening CAR-T cell associated toxicities. One of these toxicities is immune effector cell associated neurotoxicity syndrome (ICANS). ICANS usually displays a central phenotype, while peripheral neuropathy is quite rare [93] (see also the following paragraph 4 on immune-mediated toxicities).

## 4. Immune-Mediated Toxicities

### 4.1. Common Forms of Central Nervous System (CNS) Neurotoxicity after ICI Therapy

Encephalitis/meningoencephalitis usually presents with fever, headache, emesis, altered mental status, seizures. Aspecific inflammatory changes are evident in brain magnetic resonance imaging, and also electroencephalography reveals aspecific theta slowing abnormalities. Cerebrospinal fluid (CSF) assays commonly show albumino-cytologic dissociation [94,95]. Extensive CSF paraneoplastic and autoimmune antibody assays should be negative to rule-out autoimmune encephalitis or cerebellitis [96].

Vasculitis, mostly in the form of giant cell arteritis or isolated retinal vasculitis, have been described in patients receiving up to 15 treatment cycles of anti-PD-1 therapy [97]. Laboratory testing of erythrocyte sedimentation rate and C-reactive protein is warranted, while biopsy of the temporal artery is the mainstay to confirm a diagnosis of giant cell arteritis. Hypofluorescence in the area of the active lesion in fractional anisotropy is in keeping with retinal vasculitis [98].

Cases with de novo CNS demyelination, resembling multiple sclerosis, or with anti-aquaporin-4 antibody-positive neuromyelitis optica spectrum disorder have rarely been reported after PD-1 exposure [99]. On the other hand, anti-CTLA-4 therapy has been associated with progression of radiographically isolated syndrome into clinically definite multiple sclerosis [100].

Steroid treatment-responsive neuro-ophthalmological IRAEs in the form of unilateral or bilateral optic neuritis have rarely (1%) been described after 2–12 weeks of exposure to ipilimumab [101,102], pembrolizumab [103], nivolumab monotherapy [104] or combined with a peptide vaccine [105], atezolizumab [106] and durvalumab [107]. Cranial nerve palsies, affecting the oculomotor, abducens and facial nerves have also been rarely reported after up to 13 months of ICIs treatment initiation [108]. Finally, very late neuropsychiatric effects to include cognitive and mood disorders are also seen in a few ICIs-exposed cancer patients, particularly those with other collagen disease comorbidities [109].

### 4.2. Common Forms of PNS Neurotoxicity after ICI Therapy

Immune-related myositis (irMyositis), mostly affecting elderly male cancer patients soon after the initiation of ICI treatment [110], appears to be the most common neuromuscular toxicity of anti-PD-1/anti-PDL1 and anti-CTLA-4 immunotherapy [111,112]. The clinical phenotype of irMyositis does not significantly differ from the typical myositis and is characterized by diffuse myalgias and weakness in the back and proximal muscles of the pelvic girdle, and reduced tendon reflexes [113,114]. Ptosis, ophthalmoparesis, facial weakness and involvement of bulbar muscles is present in about 50% of ICIs-exposed patients [110,115]. Respiratory or cardiac muscle involvement is seen in severely affected patients and requires admission in an intensive care unit (ICU). Laboratory testing with increased CK levels up to fivefold over normal and recording of a typical myopathic pattern in needle electromyography are strongly supportive of irMyositis. A definite diagnosis is established with presence of necrotizing inflammatory myopathic changes at muscle biopsy [115]. However, not all patients present abnormal findings in muscle sampling mostly because patients have already been started treatment with corticosteroids immediately after a tentative diagnosis of irMyositis has been set.

Immune-related myasthenia gravis (irMG), although less frequent than irMyositis, represents the most common and life-threatening neuromuscular toxicity after ICIs exposure. The co-occurrence of irMG and irMyositis significantly increases the risk of myasthenic crisis with need of ICU admission [116]. irMG typically manifests with fluctuating muscle weakness involving ocular, bulbar and/or respiratory muscles either de novo or as a relapse upon a pre-existing MG after an average of 6 weeks of ICIs therapy initiation [117]. Confirmatory tests for irMG include pyridostigmine or edrophonium challenge, single-fiber electromyography, and positive serum acetylcholine receptor antibody assays [115].

Although ICIs are generally less neurotoxic to peripheral nerves than conventional chemotherapy, peripheral neuropathies can also occur in the form of immune-related demyelinating polyradiculoneuropathies or axonal sensory neuropathies [114,115]. Up to 8% of patients develop immune-related demyelinating polyradiculoneuropathies after exposure to 3-4 courses of PD-1/PDL-1 therapy [118]. Immune-related demyelinating polyradiculoneuropathies presents with acute or subacute onset of numbness/paresthesia, muscle weakness, neuropathic pain in distal extremities and cranial nerve involvement with bulbar symptoms and dyspnea. Albumin-cytological dissociation in CSF is typically seen, while an electrodiagnostic confirmation of demyelinating polyradiculoneuropathies requires a marked motor conduction slowing and F wave prolongation, as also chrono-dispersion. Antiganglioside antibodies assays are of low diagnostic sensitivity [119].

Symptoms of sensory peripheral neuropathy after ICIs therapy clinically resemble the typical peripheral neurotoxicity after exposure to conventional chemotherapy with taxanes or platinums. In about 1% of exposed patients, there is de novo evidence of distal positive and negative sensory symptoms in a stocking-and-glove distribution, areflexia and proprioception changes usually occurring after commencing 3–7 cycles of ICIs [114]. NCS are compatible with an axonal sensory neuronopathy with reduced or absent amplitudes of sensory action potentials in examined nerves, mostly sural and dorsal sural nerves. Nonetheless, the neuropathy seems to have a benign course with quite low occurrence of treatment-emergent grade 3 neurotoxicity (0.3%) which generally recover soon after ICIs discontinuation even without treatment with corticosteroids [114].

### 4.3. Neurotoxicity after CAR T Cell Therapies

One of the main limitations of CAR-T cell therapy is ICANS, which could be severe and life-threatening, as already mentioned. ICANS can develop in up to 70% of exposed patients [120] as opposed to the much lower frequency of ICIs-related neurotoxicity that affects less than 10% of patients [121]. Typically, ICANS develops as a very early side effect of CAR-T cells within 3 to 10 days after therapy initiation [122]. The clinical phenotype includes confusion, headache, inattention, language deficits, focal neurological deficits, or encephalopathy that can be severe and life- threatening in up to 30%. Severely affected patients exhibit diffuse cerebral edema, comatose state and seizures [123]. Younger patients and those with preexisting neurological and other medical conditions, as also those with increased tumor burden and high intensity lymphodepleting therapy, are more liable to manifest ICANS [124]. Most times ICANS is accompanied by a cytokine release syndrome, the other most frequent irAEs on CAR-T therapy, but sometimes it may appear as isolated presentation. Confirmatory laboratory tests include high serum lactate dehydrogenase and cytokine levels, thrombocytopenia and increased inflammatory markers [124]. Neuroimaging is not specific in most of the cases and white matter changes as well as sulcal effacement are only evident in comatose ICANS patients with diffuse cerebral edema [125]. Electroencephalography reveals diffuse slowing with recording of theta-delta rhythms [126].

### 4.4. Possible Mechanisms of Novel Immune-Mediated Neurotoxicity

The pathogenic mechanisms underlying neurological immune-related adverse events (NirAEs), and their low incidence in comparison to other immune-related toxicities in other systems, remain largely unknown. However, several factors and mechanisms have been proposed, although it is still vaguely defined if ICI drugs act locally in the nervous system or as a consequence of the transmigration upon the peripheral activation of the immune system [127].

**Figure 1:**
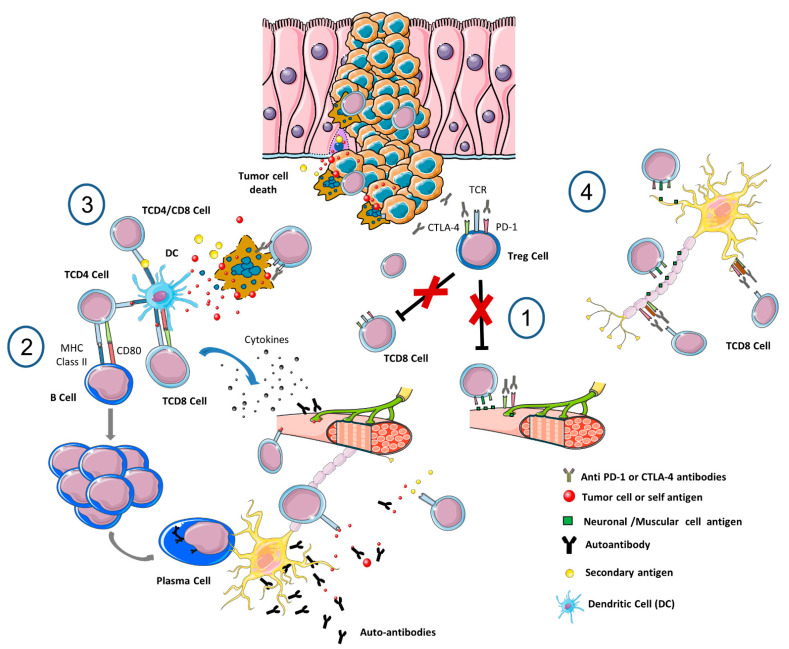
Potential pathological mechanisms underlying neurological immune-related toxicities (NirAEs). A: Hypothesis under ICI. (1) ICIs may induce Tregs reduction and shift the balance towards immune tolerance loss and immune toxicity development (2) The similarity between a neoantigen and a self-antigen might induce T- or B-autoreactive cells wrongly directed against self-antigens (molecular-mimicry) (3) Cytotoxic CD8+ T-cells evoke tumor cell death but also non-transformed bystander cells death. The antigens released by both types of cells (antigen spreading) might be ingested and processed by antigen-presenting cells (APCs) priming new T- or B-cells leading to a autoimmunity reaction (4) PD-1 and CTLA-4 antibodies could recognize their target molecules expressed by non-hematopoietic cells, like in the nervous system, and induce a local injury through antibodies or T-cell cytotoxicity mechanisms. *Modified from Vilariño N* et al. *2020* [127].

First, as in other cytotoxic treatments, the anatomical barriers, the peculiarities of other resident cells (such as glia and microglia) to generate a differential microenvironment, and the particular lymphatic system structure of the CNS might partly explain this differential ratio of NirAEs and immune-related adverse events in other systems. Second, several theoretical mechanisms can be involved depending on the autoimmune-induced disorder (Figure 1).

Breakage of the immune tolerance by the depletion of regulatory T (Tregs) cells [128], the loss of B-cell tolerance and subsequent changes in the cytokine profile. Both Tregs and B cells present CTLA-4 and PD-1 receptors; and proinflammatory cytokine profiles [129], and/or development of serum autoantibodies [130] have been identified in ICI-treated patients. The established role of Tregs depletion in experimental autoimmune encephalomyelitis and MS might also support the latter point of view [131].

Molecular mimicry and cross-reactivity between tumor and self-nervous system antigens is supported by multiple experimental and clinical studies [132]. The self-antigens might be released when tumor and peritumoral tissues are damaged by the activated immune cells and other concomitant treatments. A paradigmatic example of cross-reactivity might be some paraneoplastic syndromes [133].

In contrast to cross-reacting antibodies, epitope spreading might be induced by the release of secondary tumor and non-tumor antigens initially not recognized by the firstly activated T cells [134]. These antigens prime B and T cells causing a new immune-mediated response. This hypothesis is exemplified in the so-called immunogenic cell death, triggered by some conventional anticancer treatments, but relevant evidence on the contribution of NirAEs is lacking.

Recognition of immune checkpoint receptors in the nervous system as an off-target effect. These receptors are also expressed in astrocytes and neurons, among other non-immune tissues to facilitate self-immune-tolerance. This mechanism is well established in ICI-induced hypophysitis, but has an uncertain role in other NirAEs [127].

Finally, ICI treatment can unmask preexisting and still subclinical autoimmune reactions against neuronal antigens or paraneoplastic syndromes [132]. In addition, a genetic background, mainly the HLA, and the microbiome composition although are only probably related with the stochastic immune-related adverse event risk, there is no strong evidence of its involvement in NirAEs.

**Figure 2:**
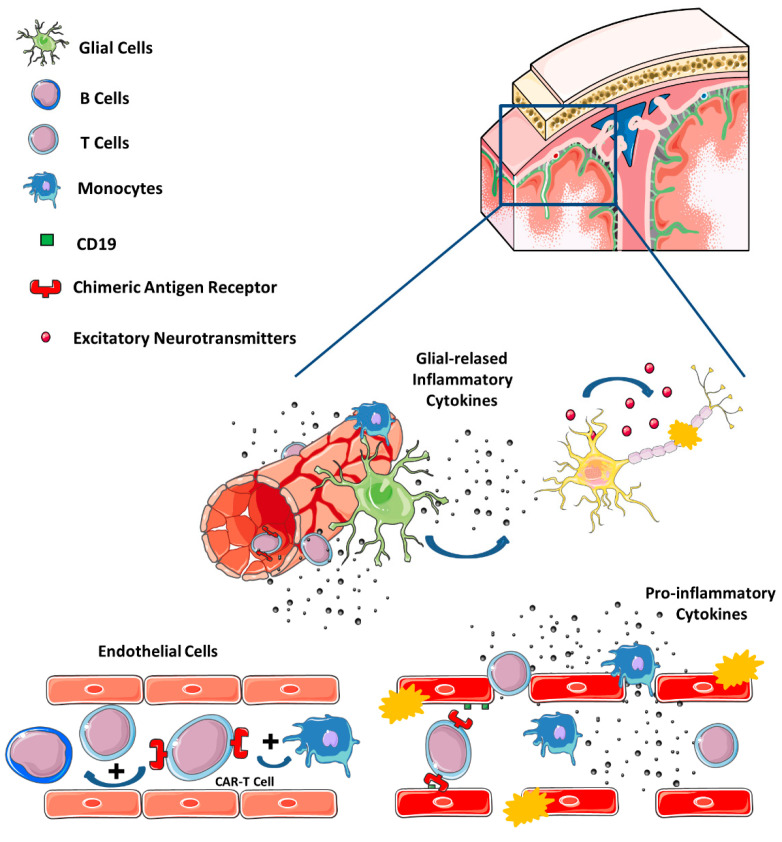
Mechanisms related with CAR-T cells. CAR-T cells induce a proinflammatory secretory phenotype of other blood circulating T and B cells or/and monocytes. This inflammatory state activates the endothelial cells and increase the permeability of the blood-brain barrier. This phenomenon allows the exposition to high concentrations of cytokines and the transmigration of activated immune cells on the cerebrospinal fluid. This inflammatory damage to the endothelial cells and glia enhances production of specific glial cytokines and stimulates the release of excitatory neurotransmitters that can induce excitotoxic damage. In addition, endothelia cells that express CD19 can also be directly damaged by CAR-T cells. *Modified from Vilariño* et al. *2020* [127].

For CAR-T treatments, as already stated, the main neurological adverse event is ICANS. This syndrome is a consequence of the cross talk between CAR and other non-neoplastic cells (like T and B cells, monocytes, endothelial, and stromal cells), leading to the release of proinflammatory chemokines and cytokines. This inflammatory state and the activation of endothelial cells increases the permeability of the blood–brain barrier, facilitating the exposition of the cerebrospinal fluid to high concentrations of systemic cytokines and immune cells. This phenomenon, in turn, can induce brain vascular pericyte and endothelial stress, production of specific central nervous system cytokines, and elevation of NMDA receptor agonists—such as glutamate and quinolinic acid—that can induce excitotoxicity [122,135]. In addition, cells that express CD19 antigen in the neurovascular unit have been identified, specifically in pericytes and smooth muscle cells, supporting an on-target mechanism for neurotoxicity [136], as shown in Figure 2.

On the other hand, other Authors have suggested that heterogeneity in the cellular and molecular features of CAR T cell infusion products contributes to variation in efficacy and toxicity: a rare cell population with monocyte-like transcriptional features was identified in axicabtagene ciloleucel CD19 CAR-T patients with high grade neurotoxicity [137]. Finally, the antigen binding domain’s affinity to its target epitope [138] and the immunogenicity of the antibody fragments [139], together with the hinge and transmembrane domain [139,140] or the co-stimulatory domains [141] may contribute to the level of CAR activation and risk of toxicity.

### 4.5. Treatment or Potential Treatment Strategies

Probably, the first step for a successful treatment is the early detection of NirAEs from any immune-mediated cancer treatment modality, to minimize the severity and prevent the amplification of the deleterious immuno-inflammatory response. This also implies to rule out any other metabolic, infectious or cancer-related conditions. Thus, a close clinical and laboratory as well as neuroimaging and neurophysiological monitoring of these patients is strongly advised [142,143]. However, the identification of risk factors and possible biochemical markers has to improve, also for the rarity of ICI-induced NirAEs.

The design of specific treatments is impaired by the lack of well-established pathogenic mechanisms, especially in ICI complications, and because the same syndrome may be yielded by concurrent mechanisms in different patients (such as autoantibodies, T-cell infiltration). Moreover, extrapolations done from pathological mechanisms under similar syndromic primary autoimmune-diseases may not be representative of all the cases.

The management of ICI-induced NirAEs is based on the ICI withdrawal and the prompt administration of corticosteroids. Empirical evidence has shown that oral schedules of prednisone 0.5–1 mg/kg/day for low grade toxicities, and intravenous methylprednisolone 1 g/day (during 3–5 days) for severe toxicities are useful to successfully manage the neurologic complications, even in cases of immune-related demyelinating polyradiculoneuropathy. In the latter scenario, intravenous immunoglobulins can be simultaneously added. Patients with refractory symptoms might undergo several lines of treatment until clinically relevant response occurs: corticosteroids, as first line, and immunoglobulins, plasma exchange, rituximab, or other immunosuppressive treatments, such as cyclophosphamide, methotrexate, infliximab, as second line [143]. However, the efficacy of these second-line treatments is limited, and seems to improve only 20% of patients [144]. Corticosteroids have to be slowly tapered, over 2–3 months, to avoid early relapses [111,114].

If the pathogenic mechanisms for each NirAE will be elucidated in the future, other targeted therapies, such as anti-integrin mAb, interleukin inhibitors or rituximab, can be considered as first treatment with the intention to preserve the efficacy of ICIs.

In CAR-T neurotoxicity, the mainstay treatment is the supportive care and corticosteroids. Tocilizumab, a mAb against the interleukine-6 receptor, was the first treatment approved for the cytokine-release syndrome associated with CAR-T therapy. However, tocilizumab has limited efficacy to prevent or treat ICANS. Other interleukin inhibitors under investigation with capacity to cross the blood–brain barrier, like anakinra (interleukin-1), siltuximab (interleukin-6) or lenzilumab (granulocyte-monocyte-colony stimulating factor), might ameliorate the effective management of ICANS [142,145]. Additional treatment strategies are also under assessment, as etanercept (tumor necrosis factor-α inhibition), ruloxitinib and itacitinib (JAK/STAT pathway inhibition, used by the interleukine-6), dasatinib (for a T cell activation switch), defibrotide (to attenuate the endothelial cell activation), and modifications of CAR-T constructs to include suicide genes if they are over activated [146]. However, high-dose corticosteroids (dexamethasone 10 mg/6–12 h or methylprednisolone 1 g/12 h) still remains the recommended first line approach to treat moderate or severe neurotoxicity [142]. This approach has the advantage of improving the integrity of the blood–brain barrier and modulating the T-cell activity. Despite the initial reluctance to use it, subsequent studies have shown that patients receiving corticosteroids have low risk to ablate CAR-T cell expansion or affect the antitumor efficacy [147].

## 5. Chemotherapy-Related Cognitive Impairment

### 5.1. Definition and Clinical Presentation

Chemotherapy-related cognitive impairment (CRCI) in patients with non-central nervous tumors is referred as chemofog or, more commonly, chemobrain.

Chemobrain is defined as a worsening in cognitive function related to cancer treatment. In the present review, we exclusively refer to the CRCI, while several authors include in chemobrain all treatments-related cognitive impairment (e.g., radiation, surgery, endocrine therapy). Cognitive impairment in chemobrain is usually mild to moderate and becomes more noticeable once patients try to resume their normal activities. Chemobrain has mainly been studied in middle-aged women with breast cancer, hence subtle cognitive symptoms may be brought to clinical attention when patients aim to return to their jobs and social interactions. Nonetheless chemobrain has been reported, with variable incidence, in survivors of many other cancers [148,149,150,151], and is associated with a significant decline of QoL, self-confidence and independence with a severe social and economic impact [152,153,154,155].

The most affected cognitive domains are memory, executive functions and processing speed. Symptoms may include difficulty in concentrating, multitasking, learning new skills, remembering a conversation (verbal memory), recalling an image or list of words (visual memory), maintaining prolonged and focused attention span, finding the right word (tip-of-the-tongue phenomenon). Patients report feeling of mental fogginess and became unusually disorganized. They also take longer than usual to complete routine tasks and complain of distractibility [153,156,157]. The neuropsychological pattern is slightly different depending on the type of cancer and treatment. Patients affected by breast cancer struggle with short-term memory, word retrieval, concentration, and have a hard time completing tasks and learning new skills while other functions as language and visuo-constructional ability are preserved. Verbal memory impairment has been found in patients with colorectal cancer while worsening in phonemic fluency, information processing, working memory, and visuospatial abilities were found in small cell lung cancers survivors [149,158,159,160,161].

The time course of CRCI is variable. It appears more frequently at the end of chemotherapy, but patients may experience cognitive decline throughout all the course of the active treatment. CRCI gradually ameliorates in the first 3 years after treatment [155,162,163,164,165].

Chemobrain may be associated with affective disorders (anxiety, depression) secondary to a diagnosis of cancer thus generating erroneous identification between these two conditions [156].

### 5.2. Mechanisms of Chemotherapy-Related Cognitive Impairment (CRCI)

The mechanisms of CRCI in cancer survivors are not fully understood and are likely to be multifactorial.

Hypotheses on how chemotherapy induces cognitive impairment derive from in vitro and mouse models and include several mechanisms, among which is oxidative stress. Doxorubicin, cyclophosphamide and other agents are known to cause oxidative damage to neurons’ organelles (mitochondria and peroxisomes) and to DNA leading to neuronal loss. Inflammation and microglial activation seem to also significantly contribute to CRCI. Several chemotherapeutic agents, together with cytokines released from host cells and tumor tissue itself, may increase peripheral and brain proinflammatory cytokines (IL-1b, IL-6, TNF-a, IL-10) production, thus exacerbating inflammatory processes; specifically, cytokine-related inflammation was demonstrated to interfere with frontal lobe function.

Beside the direct neuronal damage caused by cytokines, it is also possible that inflammation activates microglia, which may cause further damage to neurons. Moreover, cyclophosphamide, doxorubicin, and 5-fluorouracil have been shown to prevent the neurogenesis in the hippocampus, and also cisplatin and doxorubicin interfere with synaptic function, affecting neuronal activity and plasticity.

Finally, neurotransmitter depletion secondary to neuronal damage may contribute to cognitive impairment [166,167,168,169,170,171,172,173,174,175,176].

Limited research into the cognitive effects of ICIs has been performed to date but preliminary data suggest a possible role of microglia activation [177]. Elevated levels of leukocytes and proinflammatory cytokines and the accumulation of CAR T cells may lead to neurological symptoms in CAR T cell therapies [135]. A peculiar mechanism may be advocated for taxane-related cognitive impairment. Taxanes interfere with the microtubule structures which are crucial for the formation and stabilization of spines, dendrites and axons. Moreover, the microtubule network is critical for maintaining neurotransmission. Therefore, taxanes can prevent spines and dendritic physiological arborization, thus resulting in cortical gray matter loss and can impair neurotransmission, thus interfering with hippocampal functions [178,179,180]. The heterogeneity of neuronal injury mechanism is consistent with the fact that the cognitive profile of chemobrain does not show anatomical or domain specificity but appears more diffuse with a multi-lobe subcortical profile. Accordingly, advanced MRI studies showed a widespread involvement of white matter microstructures, a global reduction of grey matter and default mode network connectivity reduction [181,182,183].

Studies aiming at characterizing the specificity of the single drugs on cognitive profile or on the anatomofunctional brain fingerprint are still scarce. Several factors may increase the risk of CRCI, including genetic predisposition (i.e., the presence of the apolipoprotein Eε4—APOE ε4—and COMT-val allele), hormonal dysfunction (due to anticancer hormonal treatments or secondary to chemotherapy or surgery), clinical conditions (pain, insomnia, fatigue) [172,184,185,186,187,188]. Susceptibility to chemobrain is also related to vascular risk, diabetes, lower education, older age and less daily activities thus suggesting a possible role for cognitive reserve [189,190].

### 5.3. Assessment and Therapeutic Approach/Strategy

Currently, diagnostic criteria and specific tests for chemobrain have not been uniformly established in clinical studies thus accounting for the large variability in the prevalence of chemobrain across different cancers and different studies [156,191].

Subjective perception of cognitive dysfunction should be evaluated with functional assessment of cancer therapy—cognitive function (FACT-Cog), which correlates with quality-of-life deterioration [186]. Besides patient complaints, neuropsychological testing is mandatory and provides objective assessments across cognitive domains. According to the International Cognition and Cancer Task Force recommendations [191] the following tests should be performed: Hopkins Verbal Learning Test-Revised (Verbal memory and delayed recall), Controlled Oral Word Association Test (speeded lexical fluency and executive function) and Trail Making Test (psychomotor speed and executive function). This core battery should be implemented with additional tests exploring functions like working memory, executive function and complex attention; therefore, the following tests may be considered: Auditory Consonant Trigrams, Letter–Number Sequencing, Paced Auditory Serial Addition Test, Brief test of attention. Cognitive evaluation should be performed longitudinally to detect the slightest changes throughout the chemotherapy cycles.

The first evaluations should be ideally performed before any treatment (e.g., surgery and radiation) and then before the first chemotherapy cycle. Notably, neuropsychological tests were designed and validated for other conditions and may have low sensitivity and specificity to detect the relatively subtle cognitive changes experienced by cancer survivors therefore a mismatch between cognitive complaints and normal scores is not unusual [192,193,194]. Moreover, functional MRI studies suggest the activation of compensatory mechanisms by recruiting additional brain regions to maintain cognitive performance. This overactivation of the brain may justify the cognitive complaints in daily life despite normal performance at neuropsychological testing [195].

Screening for depression symptoms should also be performed in these patients. Imaging, molecular or serum biomarkers or predictors of chemobrain are still missing [196].

At the moment, no standard of care is available for chemobrain and treatment options are limited. A reasoned approach may consist of a combination of strategies including reassuring the patient, treating comorbidities, encouraging physical activity and providing behavior interventions and cognitive training. Physical activity demonstrated improvement of cognitive symptoms in breast cancer survivors and preservation of hippocampal neurogenesis in rat models. However, few studies addressed this type of intervention without consistency in training protocol and neuropsychological evaluation [197,198]. Cognitive training showed positive results on symptoms but variable improvement on test battery scores [199,200]. Pharmacological approaches have been identified both as preventive (to be assumed together with chemotherapy) and as symptomatic treatment (to be assumed once cognitive dysfunction has been established). Pharmacological strategies may include central nervous system stimulants (e.g., methylphenidate and modafinil), antidementia drugs (e.g., donepezil, memantine, and ginkgo biloba) but their efficacy in randomized clinical trials has not been yet established and their use in clinical setting remains limited [172,201].

## 6. Conclusions and Future Perspectives

Many neurological adverse events, older and newer, have entered the everyday clinical practice of health-care professionals entrusted with the care of oncological patients, and specifically cancer survivors.

Despite many efforts in the last few decades to ameliorate recognition, pathogenic knowledge and treatment of neurological sequelae, there are still many unmet clinical and scientific needs. To provide patients and treating physicians with robust mechanistic background and effective solutions to manage all these clinical entities, an interdisciplinary effort is warranted. Towards the latter view, preclinical and clinical scientists should cooperate to design both preclinical experiments and clinical trials with the strongest methodological approach possible. The collaboration among different specialists (e.g., oncologists, neurologists, pain therapists) and general practitioners [202] is crucial and should aim at a better knowledge and management of neurological adverse events through the development of common and joint strategies to cope with them. Moreover, while we are still waiting for neuroprotective drugs [203,204,205], a better understanding of pathogenesis will lead to a tailored treatment that will help to obtain the best benefit for any individual patient.

Therefore, an alliance among bench and bedside will enable to ascertain pathogenic issues and empower drug development to treat these conditions. The collaboration of preclinical and clinical research, relying on a multidisciplinary and interdisciplinary approach, can flourish thanks to international large initiatives that connect experts from all over the world, as exemplified by the Toxic Neuropathy Consortium of the Peripheral Nerve Society which built the ideal environment to pave the way to new line of research in this field [204].
